# Interleaved Honeypot-Framing Model with Secure MAC Policies for Wireless Sensor Networks

**DOI:** 10.3390/s22208046

**Published:** 2022-10-21

**Authors:** Rajasoundaran Soundararajan, Maheswar Rajagopal, Akila Muthuramalingam, Eklas Hossain, Jaime Lloret

**Affiliations:** 1 School of Computing Science and Engineering, VIT Bhopal University, Kothri Kalan 466114, India; 2 Department of ECE, Centre for IoT and AI (CITI), KPR Institute of Engineering and Technology, Coimbatore 641407, India; 3 Department of CSE, Centre for IoT and AI (CITI), KPR Institute of Engineering and Technology, Coimbatore 641407, India; 4 Department of Electrical and Computer Engineering, Boise State University, Boise, ID 83725, USA; 5 Instituto de Investigacion para la Gestion Integrada de Zonas Costeras, Universitat Politecnica de Valencia, 46022 Valencia, Spain

**Keywords:** wireless sensor networks, security, wireless honeypots, Medium Access, wireless medium, attacks

## Abstract

The Wireless Medium Access Control (WMAC) protocol functions by handling various data frames in order to forward them to neighbor sensor nodes. Under this circumstance, WMAC policies need secure data communication rules and intrusion detection procedures to safeguard the data from attackers. The existing secure Medium Access Control (MAC) policies provide expected and predictable practices against channel attackers. These security policies can be easily breached by any intelligent attacks or malicious actions. The proposed Wireless Interleaved Honeypot-Framing Model (WIHFM) newly implements distributed honeypot-based security mechanisms in each sensor node to act reactively against various attackers. The proposed WIHFM creates an optimal Wireless Sensor Network (WSN) channel model, Wireless Interleaved Honeypot Frames (WIHFs), secure hash-based random frame-interleaving principles, node-centric honeypot engines, and channel-covering techniques. Compared to various existing MAC security policies, the proposed model transforms unpredictable IHFs into legitimate frame sequences against channel attackers. Additionally, introducing WIHFs is a new-fangled approach for distributed WSNs. The successful development of the proposed WIHFM ensures resilient security standards and neighbor-based intrusion alert procedures for protecting MAC frames. Particularly, the proposed wireless honeypot methodology creates a novel idea of using honeypot frame traps against open wireless channel attacks. The development of a novel wireless honeypot traps deals with various challenges such as distributed honeypot management principles (node-centric honeypot, secretly interleaved-framing principles, and interleaving/de-interleaving procedures), dynamic network backbone management principles (On Demand Acyclic Connectivity model), and distributed attack isolation policies. This effort provides an effective wireless attack-trapping solution in dynamic WSNs. The simulation results show the advantage of the proposed WIHFM over the existing techniques such as Secure Zebra MAC (SZ-MAC), Blockchain-Assisted Secure-Routing Mechanism (BASR), and the Trust-Based Node Evaluation (TBNE) procedure. The experimental section confirms the proposed model attains a 10% to 14% superior performance compared to the existing techniques.

## 1. Introduction

Wireless Sensor Networks (WSNs) are diversely used for significant applications such as industrial automation, animal-tracking systems, ocean-monitoring systems, defense support systems, rural surveillance systems [1], and others. Usually, WSNs are created with numerous wireless sensor nodes placed around the required geographical locations. The sensor nodes are deployed on land surfaces, water surfaces, and other hostile regions to sense the ecological data from various objects.

The sensed data are processed and communicated to respective sink nodes through multi-hop-routing procedures. The data on the wireless medium are more vulnerable compared to dedicated wired channels. WSNs need optimal protection via channel security for their application-specific network architectures. As wireless sensor nodes operate under limited internal resources with respect to their memory, battery source, and processor efficiency, the security algorithms must provide efficient and lightweight solutions against unexpected channel attacks. Secure wireless data communication is essential against both active and passive attackers. The intrusions and attacks that infiltrate into data sequences on air create opportunities for data theft, identity theft, packet drops, wormholes, sinkholes, and other functional irregularities in WSNs. Attacks on wireless channels are more frequent than on wired channels. In the absence of steadfast secure servers and protected channels, the wireless sensor nodes meet open threats to live data transmissions. The technical contributions of global research are growing and thereby enabling the construction of flexible network security models for WSNs. Generally, the current wireless security models use cryptography techniques, key distribution models, blockchain technology, Intrusion Detection Systems (IDS), and channel-aware-data-hiding mechanisms.

The dedicated wired network infrastructures use firewalls, secure private network policies, centralized IDS engines, honeypots, and key distribution servers to handle attacks efficiently. Among these security models, honeypots are a collection of decoy network systems positioned among legitimate network resources. The honeypot security mechanism provides a virtual system trap with fake utilities to attract both internal and external attacks. The honeypot environment receives the incoming events and analyzes the features to decide the attacks. Here, the attackers may utilize and harm the fake resources. The results generated from honeypot engines help to enrich the network security policies gradually.

Honeypots are classified under various categories as highly interactive models, lowly interactive models, research models, and production models. Unlike wired network security models, wireless networks need more flexibility in their security layer functions. Particularly, a WSN requires completely distributed and lightweight security mechanisms that are controlled at each sensor node. The proposed WIHFM is aimed at developing a distributed honeypot environment in each sensor node to secure wireless mediums. According to the proposed idea, each sensor node holds fake internal resources to trap the attackers. The proposed model has been created for developing lightweight honeypot resources in each node with IHF contents. In this newly developed environment, the attacker trap has been created by a WIHFM that is shared among legitimate data frames on the wireless channel.

The wireless MAC (802.11) policies and legitimate data frames are vulnerable to attacks. The combination of node-centric honeypot engines and the IHF traps increases the possibility of attracting various attacks from other sensor nodes. The currently accessible secure data communication models are widely anticipated and estimated by intellectual attackers. Regarding the choice of WSN security solutions, Mezrag et al. [2] proposed a lightweight energy-efficient cryptography scheme for secure data communication. In this work, secure data communication was achieved through Elliptic Curve Cryptography (identification-based cryptography techniques in the clustered WSN environment). Additionally, this scheme was assisted with distributed key distribution models. In this manner, the existing security principles stated that the identity model assured protection against different types of attacks in a WSN.

On the other hand, this technique was not enabled over random WSNs with deeply analyzed results. Guimaraes et al. [3] compared and analyzed various security protocol solutions and channel protection models for WSNs. In their analysis, the security solutions were identified as public key cryptography techniques, private key models, message authentication codes, secure hash codes, and others. The experimental details of this work are revealed through power consumption analysis, memory complexity analysis, and time complexity analysis exercises. Similarly, Sharma et al. [4] and Bhushan et al. [5] described the details of wireless security challenges and security protocols suitable for WSNs. Based on notable technical contributions, the particular type of WSN architecture and application-specific network model should be assisted by optimal security features. In this stream, Majid et al. [6] provided the details of WSN application models and security solutions. Han et al. [7] proposed genetic algorithm frameworks for enabling trust-based secure-routing mechanisms.

Correspondingly, this technique targeted the benefit of energy-aware-routing principles. Based on security concerns, Huanan et al. [8] shared vast scopes and information on the security benefits and applications of WSNs. As many studies and technical surveys on secure data communication reveal the current solutions and limitations against various attacks, the attack types are identified as basically active and passive. In this case, the denial of service, data modification, masquerading, channel interruption, misrouting activities, and other live suspicious actions are categorized under active attacks. In contrast, eavesdropping and unauthorized traffic-analyzing procedures are classified under passive attacks. In this regard, Keerthika et al. [9] described various types of malicious events such as physical attacks, link layer attacks, routing attacks, SYN-flooding attacks, smurf attacks, session-stealing attacks, and data security attacks according to the respective layers. The comprehensive understanding and comparative descriptions of various attacks inform the protection measures towards the data protection challenges in WSNs. In addition, this work suggested appropriate countermeasure techniques for different attacks.

Generally, the application of WSNs decides the levels of security features to be configured in the real-time conditions. In this case, Majid et al. [6] deeply analyzed the growth of WSNs regarding their support for industrial revolution practices. According to the analysis of WSNs’ application domains, a technological growth is expected in Internet of Things (IoT)-based solutions and other control systems. Particularly, the application of WSNs spreads through intelligent military infrastructures, health-monitoring systems, home automation systems, smart city management systems, agriculture automation systems, intelligent transportation systems, and other industrial control systems. Based on the required WSN’s model, the security policies are differently configured against expected attacks.

At the research stage, security concerns are seriously evaluated, and various solutions are proposed. Salau et al. [10] described the data security measures taken towards the protection of radio channel communications against attacks. According to this description, data security measures are expected to be taken in accordance with integrity, confidentiality, and authentication policies. On the same ground, the network’s stabilization must be assured in terms of availability. Almesaeed et al. [11] proposed channel protection and energy regulation schemes among Sybil attackers in WSNs. Sybil attackers mainly target the identities of sensor nodes. Notably, this type of attack manipulates numerous fake identities of a sensor node to damage the network reputations.

The channel protection schemes proposed in this work are equipped with node validity schemes, secure channel profile-sharing principles, signal strength indicators, and cluster management principles. Similarly, a few research methodologies found possible solutions to provide secure data transmission solutions under IoT-based WSNs [12]. Anyhow, the strategies used for promoting such data transmission solutions to ensure secure WSNs in a challenging environment are limited.

Wang et al. [13] found a correlation between the pandemic timeline and the attacks generated among IoT networks. This work analyzed critical security problems of the IoT environment through honeypot principles. According to the network architecture, this scheme deployed more than 450 honeypot engines around various geolocations. This work evaluates a real-time botnet attack detection mechanism using distributed honeypot architecture at different locations. The idea behind this honeypot model is to expose the practical possibilities of real-time botnet classification procedures. In this regard, the methodology has been designed to model numerous botnet attacks into honeypot systems. In accordance with this observation, this work finds the fragmental impacts of botnet activities and statistical findings on the systems’ performance. The continuation of honeypot-based security models is more useful for developing real-time trap systems in order to improve security practices.

In the same manner, Lygerou et al. [14] proposed distributed honeypot models for securing android devices. As this work focused on cellular network security principles, the distributed honeypots were configured for operating mobile devices (on an android platform). Similarly, the proper identification of IoT protocols interacting with mobile devices assures the actual benefit of distributed honeypot systems. In this case, the IoT protocols and the security features were analyzed for message-queuing protocols, constrained application protocols, and low-power and lossy network protocols. Mostly, the honeypot security principles are initiated from conventional data analysis tactics for detecting the attacks. For improving the efficiency of standard honeypot models, machine leaning and deep learning algorithms are widely applied in WSNs.

On the basis of motivation, Veluchamy et al. [15] proposed deep learning-based-honeypot-modelling strategies. Particularly, this approach applied deep reinforcement-learning practices to understand and detect DoS attacks at run-time. According to this approach, deep neural network functions were created to validate the internal and remote requests belonging to the network. The strategy of this work continued to compute deep score values (Q-Matrix) for network events and stated them as rewards. The experimental platform of this work initiated internal DoS events, external DoS events, website attacks, botnet attacks, and brute force attacks against deep learning-based honeypot systems. Nevertheless, a lack of system flexibility is identified in this dedicated network scenario.

Considering the computation load and time complexities, Acosta et al. [16] developed lightweight reactive honeypot principles for improving the security features of cyber deception models. In general, cyber deception is the strategy used for tricking the attackers into fake resources (honeypot). As discussed earlier, the network architecture expects suitable security functions through static models or dynamic models. The honeypot is the main security provision to assure security traps and collect the event data within them. In this case, the static model honeypots activate and control (warn) the internal node functions based on the legitimacy of the cyber event. Considering the issue, this lightweight honeypot model developed reactive node organization and channel organization principles against attacks. This mechanism ensured more optimal management routines than static honeypot approaches.

In the sequence of security considerations, Pashaei et al. [17] stated the strategy of early state intrusion detection techniques with the help of honeypots in an industrial network environment. The idea of this work was based on using Markov chain process and action-reward policies in order to observe network events continuously. The state-based honeypot event analysis model was configured to detect and isolate distributed DoS attacks and Man-in-the-Middle attacks. As mentioned, this work operated reinforcement-learning principles and Markov chain principles to detect suspicious events as quick as possible. In addition, this scheme assured its better accuracy rate based on the best reward weights according to changing event states.

As discussed in this section, various security models and honeypot frameworks are suggested to improve network security metrics. Furthermore, the honeypot-based security limitations are identified for MAC principles. As many research techniques consider network-level security challenges, the identification of channel security and MAC security provides an important and novel suggestion for innovative honeypot strategies. In this regard, this article finds the crucial limitations of the existing systems such as the lack of novel secure MAC models, distributed honeypot engines, Interleaved Honeypot Frame (IHF) solutions, and open traps in WSN. These are considered major research problems under growing attack styles and novel threats against WSNs. In this case, the newly developed security mechanism should be estimated with complex wireless channel traps. Considering these ideas, the proposed WIHFM is motivated to develop a neighbor-based distributed honeypot environment with IHF traps to attract attackers through the open channel. Mainly, the proposed WIHFM contributes the following security features for WSNs:

- On-Demand Acyclic Network Connectivity model (ODAC);
- Interleaved Honeypot-Framing Traps;
- Distributed Neighbor-based Honeypot Engines;
- Secure Hashing with Random Interleaving procedures;
- Confidential and Authenticated Channel Protection.

As per the proposed model and technical contributions, the environment of WSNs is created to optimize the network backbone and dynamic link management principles. As mentioned in the list of major contributions, the proposed model configures an ODAC for managing the associativity rules according to uncertain node links and node abilities with respect to time. According to the positioned ODAC-based network backbone and routing principles, the wireless communication shall be operated feasibly. In the next phase, wireless honeypot frames are modelled with appropriate fake data (trap frames) and internal security features among other legitimate data frames in each sensor node. The ordered inclusion of legitimate data frames and honeypot frames on the wireless channel is designed with the help of honeypot-interleaving procedures (interleaving and de-interleaving Algorithms) and randomly generated trap-slot positions. Similarly, data communication is secured by optimal channel security procedures (a secure hashing algorithm, the advanced encryption standard, and digital signature algorithms). Hence, the proposed model creates dynamic and distributed honeypot traps in each node to decoy attackers.

According to the contributions listed above, the proposed article has been systematized from Section 2, Section 3 and Section 4. In this article, Section 2 describes the materials and methods used for implementing the proposed techniques. Section 2.1 analyzes the related techniques. Section 2.2 illustrates the proposed WIHFM’s features and the technical details of the newly created IHF solutions. Section 3 explains and justifies the proposed WIHFM’s contributions with experimental results and performance comparisons. Finally, Section 4 concludes the overall contributions and provides the future scope of this novel article.

## 2. Materials and Methods

### 2.1. Related Works

Analyzing and understanding the current security solutions against various wireless channel attacks provides necessary future frameworks. In this regard, this article considers significant related contributions of recent research works. Additionally, this section provides the specific limitations of different research works and the need for a newly developed WIHFM. Pietro et al. [18] deliberated infrastructure security models and data security models for unattended WSN environments. Generally, the attacks are undertaken against sensor data’s start data modifications, false data generation, data removals, and data disclosures that affect legitimate wireless communication. According to data security models, this work discussed real-time security problems in the data streams on wireless channels. Nevertheless, the technical contribution of this model did not provide a versatile attacker detection system. As the model had been specially developed for unattended WSNs, the motivation of this work focused on channel attainment rather than crucial security benefits. Yang et al. [19] provided a perspective on the security benefits in WSNs such as location-sensitive security principles, distributed key management schemes, resilient network protection, and route classification procedures. This work identified these approaches as possible solutions against different types of attacks and node failures in WSNs. As per the contribution, this work developed the WSN security model based on random location-based key selection for protecting wireless MAC content. Additionally, this work determined counter-protection mechanisms against fabrication attacks, report interruption attacks, relocation attacks, and node compromise attacks. However, these security efforts require novel attacker-handling frameworks.

At the same time, the development of current WSN models and their variances receives notable industrial considerations. Khan et al. [20] proposed Internet of Things (IoT)-assisted flying ad hoc networks and energy-optimized-routing strategies. This work considered the obstacles of energy-efficient-routing protocols such as quality metrics, spatial movements of nodes, and dynamic network parameters. In this case, this approach proposed naturally inspired algorithms for improving the operational abilities of ad hoc routing protocols. Predominantly, the adaptation of energy-optimized ant colony optimization procedures were crucially applied to solve routing problems under uncertain network conditions. In the same manner, Singh et al. [21] found the need for IoT and WSN in healthcare applications, home control systems, multimedia applications, and smart transportation solutions. According to this work’s proposal, the current security mechanisms are not adaptive to the real-time nature of application-specific IoT environments. Based on this scientific problem, this work stated the need for more dynamic and flexible (self-reliant) security solutions for various types of networks.

Similarly, the recent advances and security requirements for WSNs and IoT were discussed by Gupta et al. [22] and Bharany et al. [23]. The crucial contributions of the existing techniques were found in the analysis of performance improvement, energy optimization solutions, security benefits, cyberspace investigations, machine learning frameworks, and blockchain-based distributed solutions. As IoT and WSN participants are mostly deployed under a distributed environment, the detailed analysis on the real-time technical issues of Gupta et al. [22] and Bharany et al. [23] provided notable information. Xiao et al. [24] developed MAC security and computation analysis models for IEEE 802.15.4 WSNs. The analysis models of this work accounted for the various MAC vulnerabilities, security principles, and platform-based overhead issues for personal area sensor network environments. According to the analysis, the mentioned article enquired about security-provisioning modes, layer-based vulnerabilities, attack types, security suites, and network adaptability.

Here, the attacks such as replay attacks, nonce attacks, and routing attacks were investigated under WSN conditions. On the other hand, the need for improvised solutions was unexploited in this development area.

Boyle et al. [25] and Karlof et al. [26] described various security architectures that can be provided for WSNs. Both articles show potential security possibilities for a wireless medium against Denial of Service (DoS) attacks, data injection attacks, identity misuse attacks, and packet-dropping attacks. Particularly, the latter work discussed link-layer attacks and security mechanisms for IEEE 802.11b and mobile communication platforms. Considering the security frameworks, these works identified excessive message overloads and processing overloads under tiny security prototype models. These works deliberately extracted the basic needs of well-defined wireless security frameworks against channel attacks. Yet, these analysis models are distorted with respect to providing unpredictable security solutions for WSNs. Similarly, Yadav et al. [27] discoursed the basic technical aspects of MAC models and communication tactics for WSNs.

Singh et al. [28] proposed redundant source management techniques and host-based IDS techniques for securing WSNs. In this regard, this scheme evaluates the possibilities of brute force attacks, key-breaking attacks, and authentication vulnerabilities. In addition, the article ensured that the need for identifying MAC-spoofing attacks and MAC duplication attacks was justified to create counter algorithms. The attack detection mechanisms implemented for building a secure WSN environment should utilize optimal sensor energy. According to the scope, the mentioned security schemes were inefficient in terms of reactive attack detection accuracy and flexible MAC communication principles. Sachan et al. [29] created energy-efficient connectivity models for mobile WSNs. This article established a novel probability channel model using network uncertainty, sensing area, node’s transmission range, and residual energy.

Elshrkawey et al. [30] and Kumar et al. [31] mentioned secure data aggregation and secure data communication practices for protecting wireless MAC layers in WSNs. Here, the first article produced individual message authentication codes for each data aggregation channel of WSNs. In this scenario, authentication codes were attached according to distributed scheduling principles concerning Time Division Multiple Access (TDMA) strategy. Similarly, the second work focused on low-energy clustering protocol and routing hierarchy model for transmitting the data. This article tried to optimize the sensor node’s residual energy to improve the quality of data transmission. Anyway, both works were limited in terms of complete distributed security solutions against channel vulnerabilities.

Khashan et al. [32] suggested lightweight encryption models for managing secure data transmission in WSNs. The limitations of conventional encryption models, authentication procedures, signature validation modules, and energy optimization techniques lead to the implantation of inaccurate security frameworks. Based on the analysis in practice, the work mentioned above-validated block chipper techniques, stream chipper techniques, and flex-crypto schemes for ensuring WSN security and increasing network lifetime. However, the adaptations of each lightweight encryption technique over different platforms were not deeply analyzed in the respective contributions.

Almansoori et al. [33] developed Secure Zebra MAC (SZ-MAC) models using elliptic curve cryptography techniques and hob-based intrusion analysis techniques. In this work, an elliptic curve cryptography engine was implemented with random key management functions for securing wireless channels. Particularly, this work deliberated clustered WSN architectures and hierarchical key distribution models to enable confidential data communication.

This work concluded that the secure SZ-MAC model confirmed optimal elimination of vulnerable entries and maximum network throughput attainment. The technical aspects of the work limit the uncertain channel handling and the development of random attacker traps.

Awan et al. [34] proposed Blockchain-Assisted-Secure-routing (BASR) mechanisms for WSNs. In this work, WSN participants were categorized as data-transmitting nodes and data aggregator nodes. According to blockchain characteristics, the consensus procedures initiated multi-path data distribution practices. This model ensured data integrity solutions using blockchain functions and confidentiality with public key cryptography functions. The results produced in the experimental section of the work were novel and highly secured against various attackers. At the same time, the flexibility of blockchain development is suitable for static WSNs. Consequently, the developed cryptography functions and blockchain structures were not flexible with respect to random WSNs.

With the same scope, Meena et al. [35] introduced secure random key management approaches and Trust-Based Node Evaluation (TBNE) approaches for the Internet of Things (IoT) environment with WSNs. In this analysis, the K-Medoid algorithm and eagle search algorithm for creating secure clusters were employed. Based on these approaches, the complete WSN was secured through distributed authentication principles. On this platform, the dynamic connectivity models and channel recreation models were not defined properly.

From the literature analysis, the proposed article finds the overall limitations of existing techniques such as the lack of node-centric IDS engines, attacker traps, MAC confidentiality, interleaved honeypot solutions, and dynamic sensor connectivity models. These are considered major research limitations to be developed to secure MAC principles. Based on providing the solutions to the above research expectations, the proposed WIHFM has been designed and developed with novel technical aspects. Section 2.2 describes the crucial features and mathematical aspects of WIHFM.

### 2.2. WIHFM

Channel-aware security principles and medium protection in WSN are creating major impacts in data communication. WSN contains a vast number of sensor nodes and independent links for establishing each channel. Wireless sessions established from each sensor node are naturally vulnerable to suspicious activities and attackers. The wireless medium needs protection in terms of secure MAC policies, secure link establishments, and secure data transmissions.

**Figure 1:**
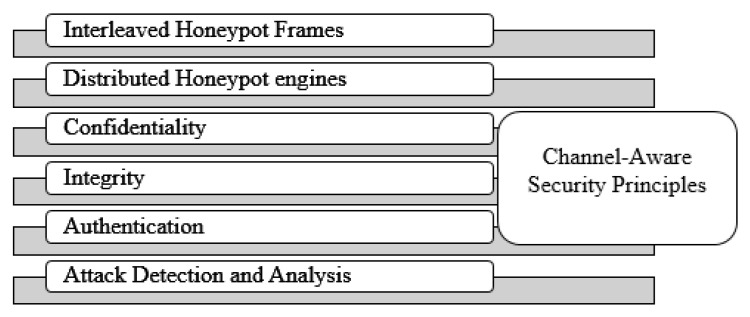
WIHFM-Security Features.

The security needs of the wireless medium can be designed and developed based on various cryptography techniques and Distributed IDS (DIDS) procedures. Figure 1 shows the proposed security features such as novel interleaved honeypot fames, node-centric distributed honeypot engines, data integrity, data confidentiality, data authentication, and DIDS procedures. The proposed channel-aware secure transmissions are expected to secure each frame before it reaches routing layer protocols. Generally, MAC frames hold three major fields such as MAC header, MAC frame body, and frame check sequence. MAC header field has been encapsulated with frame control field, session identifier, source MAC address, destination MAC address, quality parameters, variable data heap, frame protection bit, and others. Anyhow, the open wireless MAC is not secure against various channel attacks such as jamming, false data insertion, identity theft, timing disturbances, and passive channel-monitoring activities.

The secure medium places appropriate counterattacking or protection schemes against unique attack patterns. Mostly, wireless channels establish a secure MAC model using different types of cryptography techniques. The cryptography mechanisms such as public key models, private key models, signature validation models, and other integrity-checking models are employed. The provision of standard cryptography techniques are circumvented based on an attacker’s efficiency for extracting the contents of MAC frames. Here, the deployment of a novel MAC-framing solution yields unanticipated protection against channel attackers [36,37].

Thus, the proposed model develops randomly generated Interleaved Honeypot Frames (IHFs) for protecting the streams of WSN frames. Based on this idea, the network model, attacker model, suspicious event model, and proposed channel-aware security principles have been designed as follows.

#### 2.2.1. WSN Model

Let the model of WSN be assumed to have sensor node quantity denoted as ‘l’ and the geographical network area as $‘a*b’\;\left(meter^{2}\right).$ The sensor nodes will discover their neighbors and transmit the data to other nodes through wireless MAC protocol (IEEE 802.11) functions, as shown in Equation (1),

(1) $$N\left(s\right)=\left\{\begin{array}{c} l-m,\;c\geq 1\\ j,\;c<1 \end{array}\;\right.\;$$

Equation (1) shows the basic channel model of WSN. As shown in Equation (1), from $N\left(s\right)$ set of nodes, l−m nodes participate in an active communication channel [38]. At the moment, the channel quantity factor c≥1. On the other hand, j nodes are idle participants in WSN. From Equation (1), c can be expressed as follows.

(2) $$c=\left|n^{t}.dt\right|.c^{i}\;$$

Equation (2) denotes $n^{t}.dt$ as several active nodes during changing time intervals t. In this case, $c^{i}$ is the channel activity indicator used to keep +1 for live events. The WSN model has been assumed with $l(c^{i})$ active channels among overall network channels, $N\left(c^{i}\right)$. Equations (1) and (2) mainly determine the presence of wireless sensor nodes in the network. Notably, a WSN channel model determines both node availability and link availability according to time domain. Based on the Equations (1) and (2), the network assumption has been taken based on channel quality factor between multiple sensor nodes. According to that assumption, c is determined from the number of sensor nodes that are available for active communication in a channel at time t. In this regard, $c^{i}$ denotes the liveliness indication quantity for $n^{t}$ nodes at dt intervals. This component $\left|n^{t}.dt\right|.c^{i}$ is assumed to be at least 1 to observe the value of c as positive quantity. The positive quantity of c shows the presence of l−m sensor nodes in the set of total nodes $N\left(s\right)$. In this case, l−m nodes can be activated on the basis of multi-hop on-demand requests in a channel. The other j nodes are considered as either inactive nodes or dead nodes as their c value has negative magnitude.

The proposed system creates the On Demand Acyclic Connectivity model (ODAC) for enabling the logical links between active sensor nodes. The proposed ODAC model enables energy-sensitive connection establishment rules and association policies among the sensor nodes in an active channel. According to the model, $s(n^{i})$ denotes the source node, $s(n^{j})$ denotes the destination node, and the forwarding node (succeeding node) is represented as $x(n^{i})$. Based on ODAC connectivity dynamics, logical connections are established by sharing beacon messages between sensor nodes. Equation (3) clarifies the basic dynamic acyclic connectivity model [39,40]. According to the model of acyclic graph connectivity principles, the sensor nodes in the WSN are able to configure with associativity rules. In this computation, $s(n^{x})$ denotes the node taken for successive evaluation procedures for establishing acyclic connectivity links along the path between $s(n^{i})$ and $s(n^{j})$. According to the node, $s(n^{x})$ must be expected with minimal cost (diatance) to reach $s(n^{j})$. This can be rewritten as $\left\{f\left(s(n^{j}\right)+c\left(s(n^{j}\right),s(n^{x})\right\}$, and it indicates the cost function between $s(n^{j})$ and $x(n^{i})$ in the forwarding path.

(3) $$f(x(n^{i}))=\left\{\begin{array}{c} 0\;s(n^{x})=0\\ x\;,\;1\leq s(n^{x})\leq l-1\; \end{array}\;\right.$$

$$where,\;x=\min\left(s(n^{j}\right),s(n^{x}))\to \{f\left(s(n^{j}\right)+c\left(s(n^{j}\right),x(n^{i})\}$$

In this case, x denotes the minmal cost factor. Let us consider $d=f\left(s(n^{j}\right)+c\left(s(n^{j}\right),x(n^{i})))$; the ODAC model can be determined based on residual energy (joules) level $\left(n(e^{Ri})\right)$ and transmission readiness factor $\left(n(e^{ti})\right)$ of the sensor node as shown in Equation (4). The relationship between Equations (3) and (4) describes the identification of $s(n^{x})$ as successive $x(n^{i})$ associtivity in the path. In this case, d can be termed as the function of cost metric along the forwarding path.

(4) $$f(x(n^{i}))=\left\{\begin{array}{c} 0\;s(n^{x})=0\\ y\;,\;1\leq s(n^{x})\leq l-1\; \end{array}\;\right.$$

$$where,\;y=\min\left(s(n^{j}\right),x(n^{i})\to \left(d+n(e^{Ri}\right)+n(e^{ti}))$$

(5) $$d(x(n^{i}))\propto U\left(l\right)\;$$

$$U\left(l\right)-Channel\;bias\;factor$$

In the same manner, Equation (5) depicts the relationship between d and the channel bias factor $U\left(l\right)$ when $x(n^{i})$ has been continuously associated with the channel between $s(n^{i})$ and $s(n^{j}).$ This shows that the channel bias factor directly affects the manipulations of the succeeding node’s association process in ODAC mechanism. The connectivity between the sensor nodes is interrupted by different types of attackers. An attacker is a sensor node that can interpret medium data frames and other control messages. The attacker model is determined as given in Equation (6). The attacked channel frame, $Ch^{adv},$ indicates a particular target frame, $T_{fr}$, where the legitimate frame sequence $f^{i}$ is in the medium.

(6) $$Ch^{adv\_\;f}=f^{i}-\partial .sig(\nabla_{f^{i}}.I^{f}\left(f^{i},T_{fr})\right)$$

As given in Equation (6), ∂ denotes the attack-tuning function over MAC frames and $I^{f}$ indicates the frame interference or interruption function. In this equation, $\nabla_{f^{i}}$ specifies the changes in the MAC frame sequence due to attacker events. The outomes of attack interruption function can vary according to different targeted transmissions and channel frames. The model of $Ch^{adv\_\;f}$ states the impact of attackers on the channel in addition to regular channal bias conditions $U\left(l\right).$

As discussed, $I^{f}$ denotes the frame interference functions in wireless network environment. In this regard, $I^{f}$ is expressed as given in Equation (7).

(7) $$I^{f}=D^{A}.\;F^{A}.dt\;\forall \;l(c^{i})$$

**Figure 2:**
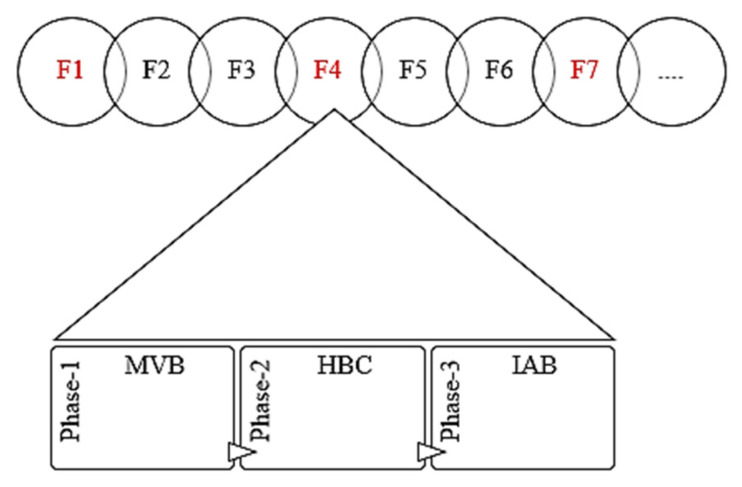
Interleaved Honeypot Frame.

Equation (7) measures the impact of adversarial data extraction functions and frame-corrupting functions $\left(D^{A}and\;F^{A}\right)$ concerning changing time intervals, dt, for all active wireless channels, $l(c^{i})$. In this case, $Ch^{adv\_\;f}$ can lead to information theft, identity theft, misrouting, false data injection, data dropping, and other channel-based attacks. Under this determination model, $\left(D^{A}and\;F^{A}\right)$ denote the regular practices of any attacker as frame data extraction and frame corruption, respectively. To protect the wireless medium and data on the channel, the proposed system creates a secure frame-interleaving approach by inserting effective honeypot frames. As given in Figure 2, the data are transferred in the wireless medium as a sequence of MAC frames through a multi-hop transmission strategy. Generally, attackers target the frames randomly or based on the information gathered from the particular data frame. The proposed system creates hidden honeypot frames that are interleaved into original frame sequences. Frames specified in red color indicate secure wireless honeypot frames inside the legitimate frame orders.

#### 2.2.2. Wireless Interleaved Honeypots

**Figure 3:**
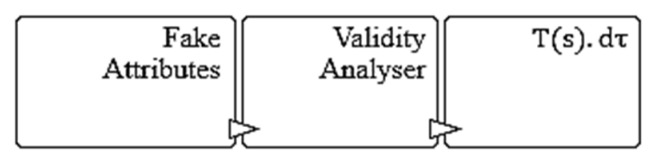
Internals of MVB.

The novel wireless honeypot-framing practices generate the chances of attackers accessing the honeypot frame instead of an original data frame. Each MAC frame sequence contains randomly inserted honeypot frames to deceive the attackers so as to make them feel as if they are accessing the original frame contents. The interleaved honeypot frame consists of an internal Message Validity Block (MVB), Honeypot Boot Block (HBC), and Intrusion Analysis and Validation Block (IVB). As shown in Figure 3, MVB provides fake frame attributes to attract the attackers and the incoming frame validity analyzer. The incoming frame is considered as an attacker’s request that is injected into the transmission sequence. The frame validity analyzer unit of MVB checks the time validity and response expectations of attacker injections. At the same time, each MVB maintains the local node’s timestamp value, $T\left(s\right),$ which indicates the time of attacker’s injection.

As mentioned in Figure 2, HBC is the snippet of boot code for initiating the internal honeypot engine functions to detect attacker events. As a preview of honeypot attack detection functions, IVB activates intrusion and attack classification procedures to create malicious logs and alert messages. Generally, the random IHF sequences (traps) are unknown to attackers.

**Figure 4:**
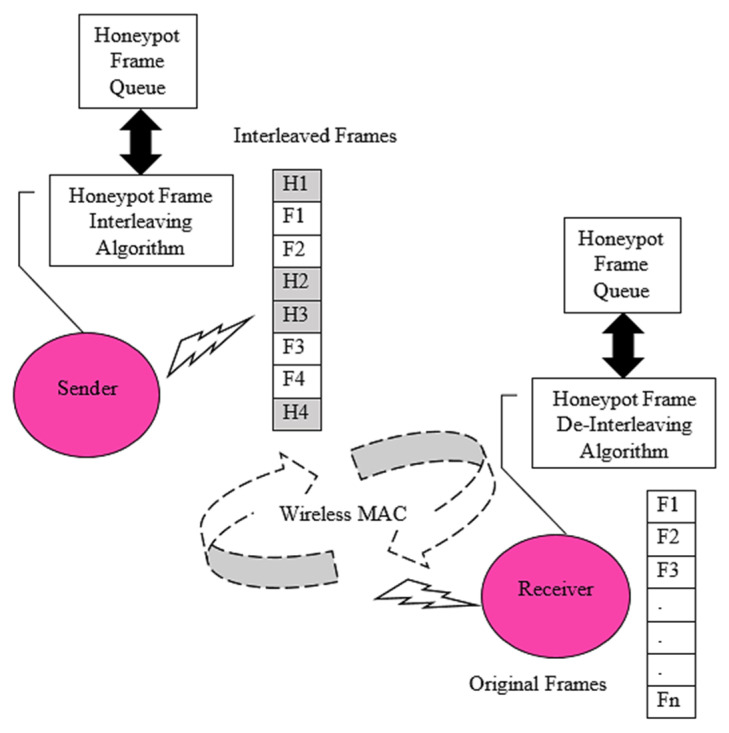
Honeypot frame-interleaving and de-interleaving process.

The proposed honeypot frames are inserted based on the frame-interleaving algorithm at the sender’s end. A sender node activates a honeypot frame-interleaving algorithm before it transmits the data on a wireless medium. As given in Figure 4, each sender-based-frame-interleaving algorithm works with an internal honeypot frame queue that holds a collection of fake frames (honeypot frames). Based on proposed honeypot insertions, wireless MAC protocol carries honeypot-inserted frame data on the channel. This novel practice creates a random trap for malicious injections. Similarly, the receiver node receives interleaved frame sequences and executes a honeypot frame-de-interleaving algorithm to obtain the original data. The secure data transmission helps to ensure resilient and flexible wireless honeypot security assurance [41,42,43,44].

The technical problem and solution to implementing random honeypot slot generation are illustrated in procedure A and procedure B. Procedure A shows random bit string generation using the sender’s internal node attributes, SHA-3 (512 Bit) computations, and random bit elector function. This approach ensures absolute slot randomness in honeypot frame insertions.

At the end of Procedure A (shown in Algorithm 1), the sender node produces these random slot identifiers as a sequence of keyed bit strings $\left(R^{i}\right)$ in a tiny tuple $Key\;\left[s\right]$. Consequently, procedure B (shown in Algorithm 2) describes the technical details of IHF productions and insertions into the sender transmission lines, $n(TX^{i})$. As discussed, each sender maintains an IHF queue, $IRQ^{i}$, to ensure secure multi-path data transmission in WSN [45,46]. In this case, $R^{i}$ are evaluated by node-centric parity bit engine to identify true cases and false cases for inserting IHF into $n(TX^{i})$. Equation (8) depicts $Key\;\left[s\right]$ in matrix format and Equation (9) shows parity bit generation for inserting IHF between original wireless frames.
**Algorithm 1.** Procedure A.
Procedure (A):
$gen:rand\left(n^{LA},s:seed\right)$
Input: $n^{L},seed,\;s:seed$ Output: random number, R 1. Get node MAC address (48 bits), $n^{LA}$ 2. Set random seed matrix $seed\;\left[i*k\right]$ 3. Computer, s:seed $\forall \;n^{i}\left(c^{i},\tau \right)$; $n^{i}-sender,\;c^{i}-channel\;id,\;\tau -Session\;id$
           
$s:seed=n^{LA}||seed\;\left[i\left(x\right)*k\left(x\right)\right]$
     
$n\_r^{i}=rand\left(s:seed\left|\left|T\right|\right|N\right)$; T−Timestamp , N−Nonce 4. Call the SHA-3 (512 Bits) function and do               $R=Sha\_3^{512}\left(n_{r}{}^{i}\right)$ 5. Start binary bit string computation,        $Rb=tr\_bin\left(R\right)$; tr_bin−Bit truncation 6. Redo for all $n^{i}$. 7. Construct binary Rb matrix, $Rb\left[i*k\right]$
8. Generate random slot bits, $R=rand\_elect\left(Rb\left[i*k\right]\right)$ 9. Store $R^{i}$ in $Key\;\left[s\right]$ iteratively 10. Recall the steps from 1 to 10.
End of Procedure (A)


**Algorithm 2.** Procedure B.
Procedure (B):
$f_{Inter}\left(R^{i},\;IHF\right)$

Input:
$R^{i}$
Output: Interleaved Honeypot Frames On Channel 1. Set
$n^{i}$ to data transmission mode 2. Get the stream of
$R^{i}$ from Procedure B 3. Set Interleaved Honeypot Frame (IHF) queue
$IRQ^{i}$
at sender
4. Do for each transmission,
$n(TX^{i})$
at sender                 
$If\left(R^{i}==p\left(1\right)\right):$            
$pop\left(IRQ^{i}\right)\;\&\;IRQ^{i}\left(count\right)--$
            
$Insert\;\left(f^{i},f^{i+1},\;IHF^{i}\right)\to n(TX^{i})$
           
$If\left(R^{i}==p\left(0\right)\right):$
            
$Insert\;\left(f^{i}\right)\to n(TX^{i})$
          
Else:
            
$n(TX^{i})\to Error$
5. Set Deinterleaver Honeypot Frame (DHF)
queue
$DRQ^{i}$
at receiver
6. Do for each reception,
$n(RX^{i})$
at receiver              
$If\left(R^{i}==1\right):$
            
$pop\left(f^{i},f^{i+1},\;IHF^{i}\right)\;\&\;push\;(DRQ^{i})$
            
$DRQ^{i}\left(count\right)++$
               
$If\left(R^{i}==0\right):$
            
$n(RX^{i})\to Receive\;Frame$
          
Else:
            
$n(RX^{i})\to Error$
7. Do for all active channels,
$C(AX^{i})$
8. Call Procedure (A) for each
$n(TX^{i})$
at
$C(AX^{i})$
9. Set the number of
$IHF^{i}$
as
$k\propto c\left(I\right)$;
$c\left(I\right)-Channel\;Independence\;Factor$

End of Procedure (B)


Based on the sequence of contiguous bit strings, the parity bits are computed for managing bit-level similarity at the sender and receiver sides. The node-centric parity-checker function finds odd parity combinations of bit strings to insert the frames.

(8) $$Key\;\left[s\right]=\left(\begin{array}{ccc} 1 & 0\;0\;0\;1\cdots & 1\\ 0 & 0\;0\;0\;1\ldots & 1\\ 1 & 0\;1\;0\;1\cdots & 1 \end{array}\right)\begin{array}{c} 1\\ 0\\ 1 \end{array}$$

(9) $$p\left(0|1\right)=\underset{p}{\sum }Key\;\left[s\right]\;\left(i*k\right)\;$$

**Figure 5:**
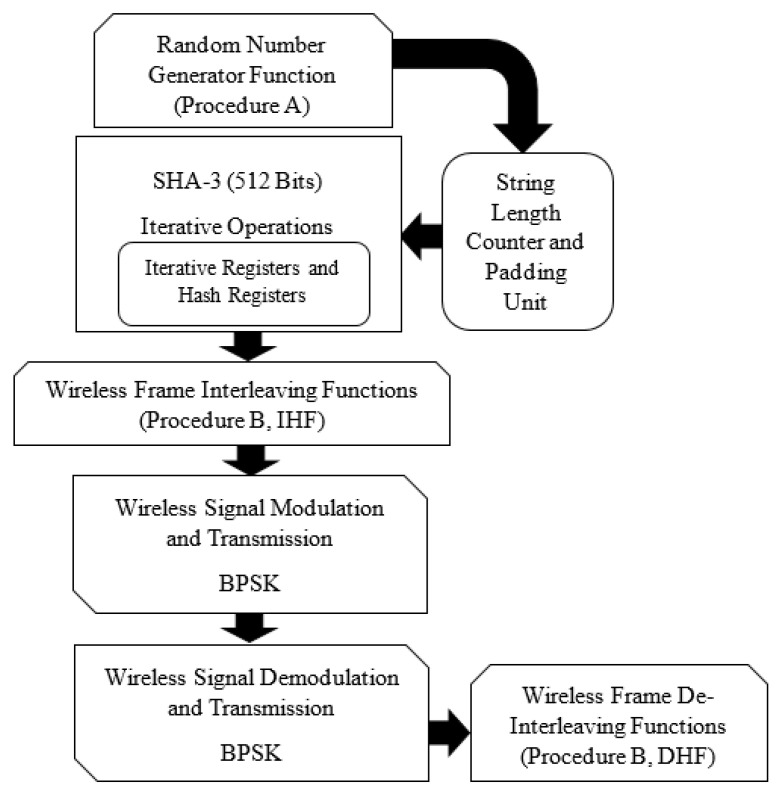
SHA-Based IHF and DHF Functions.

In WSN, there are various open channels available for sensor data transmissions. However, the active channels are being attacked by malicious activities. Here, the proposed system initiates the IHF solutions for all active wireless channels,$C(AX^{i})$. Figure 5 illustrates the flow diagram of secure hashing, random bit generations (procedure A), IHF procedures, DHF procedures, Binary Phase Shift-Keying (BPSK) modulation, and data-channeling functions.

The ICFs have a major role in acting against malicious attacks and all suspicious injections on the wireless channel. As WSN distributes the data from various sender nodes via multi-path-routing procedures, the need for sender-originated IHFs is generated for the original frame sequence. In this process, each IHF holds a random number of fake data and other lookalike network credentials to thwart the attackers [47,48].

At the same time, the interleaving and de-interleaving functions are called at the sender point and receiver point, respectively. The data-forwarding nodes and neighbors of sender and receiver are associated with exchanging the data and not extracting the contents. In the same manner, the activation of the node-centric honeypot-based DIDS engine depends on the successful malicious access to random IHF on the channel. The proposed system develops locally reactive honeypot DIDS engines in each sensor node against the attacker’s events. The proposed honeypot DIDS procedures are called due to HBC activation based on attackers’ interruptions of IHF. Procedure C (Algorithm 3) illustrates the secure wireless MAC practices based on IHF and node-centric honeypot DIDS calls.

**Figure 6:**
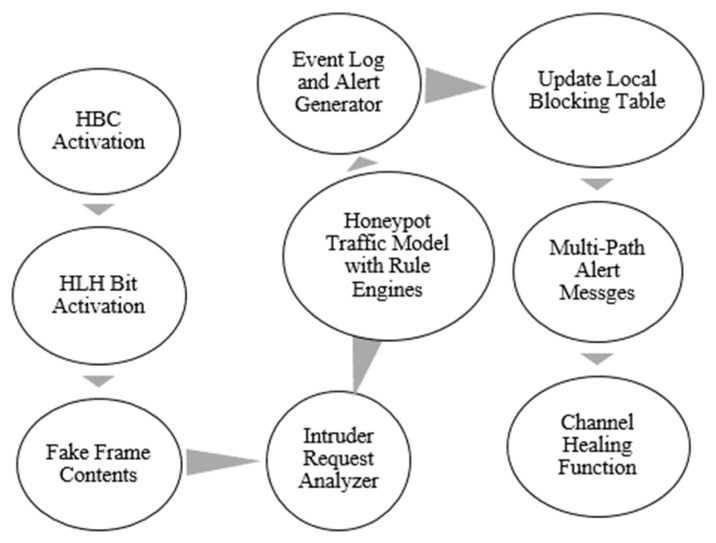
HBC and IVB.

The proposed wireless honeypot frames, $W_{H}$, are configured to expect runtime attacks and malicious intrusions and react against detected attacks based on traffic rule engines. As mentioned in procedure C, $a(mac^{i})$ indicates a successful attacker event to the IHF on the channel. According to active $a(mac^{i})$ on the wireless channel, the HBC and IVB of IHF are called to alert the immediate sink nodes of the frame on the channel. For example, $n(X^{i})$ forwards the frame sequence to $n(X^{i+1})$ through wireless MAC among attackers. Based on $a(mac^{i})$ on the channel, both $n(X^{i})$ and $n(X^{i+1})$ are alerted to call their internal honeypot DIDS engines to monitor the runtime attacks. Figure 6 illustrates HBC and IVB activations to call node-centric honeypot engines. According to Figure 6, procedure C illustrates the initialization of wireless honeypot frames, $W_{H}:Frame\left(events\right)$.

Procedure C shown in Algorithm 3 shows the active-monitoring steps to track the attacker’s access over wireless MAC frames.
**Algorithm 3.** Procedure C.
Procedure (C):
$W_{H}:Frame\left(events\right)$

Input: Attacker access
$a(mac^{i})$
Output: Attacker logs and report 1. Do for all attacker access over MAC frame sequence,
$a(mac^{i})$
         
$If\;\left(a(mac^{i},IHF^{i})\right)==TRUE):$
            Go to step 2.         
$If\;\left(a(mac^{i},IHF^{i})\right)==FALSE):$
            Go to step 3. 2. Call HBC and IVB functions (Procedure (D))          Create an alert to all neighbours,
$n^{n}$
          Make an entry in the attacker’s record,
$A(r^{n})$
3. Continue
$n(TX^{i})$
and
$n(RX^{i})$

4. Do for all active channels,
$C(AX^{i})$
5. Call procedure (A) and procedure (B) for activating each
$n(TX^{i})$
at
$C(AX^{i})$

End of Procedure (C)


Compared to other existing mechanisms, the proposed wireless honeypot principles ensure real-time data security only on active channels rather than entire channel connections. The reactive security support system provides a novel solution against various attacks with minimal energy consumption. The attack alert system for each channel works based on the attacker database and rule-based intrusion detection policies [49,50]. These DIDS policies are executed concerning the sensor node’s local decision-making functions and MAC alert functions. Based on successful HBC and IVB functions, the security internals of the sensor node call procedure D (shown in Algorithm 4). Procedure D initiates event analysis functions for each channel transmission during various active intervals. In this traffic analysis procedure, each request of various internal nodes is validated concerning $\mathrm{Rule}\;\mathrm{set}\left(a\right)$ to detect the intrusions over the channel.
**Algorithm 4.** Procedure D.
Procedure (D):
$IDS\_Engine\left(HBC\;and\;IVB\right)$

Input:
$Data[a(mac^{i})]$
Output: Suspicious events detection 1. From Procedure C (step 2) 2. Set node’s internal intrusion dataset
$I\left(f\right)$
3. Get
$events\;(a(mac^{i}))$
at node
$n^{i}$; sender or forwarding node 4. Call
$IDS\_Engine\left(HBC\;and\;IVB\right)$
at
$n^{i}$
           
$If\;\left(a(mac^{i},IHF^{i})\right)==TRUE):$
             Call IDS rule engine functions             Start
$ule\;set\left(a\right)$;
a−traffic attributes, Table 1.              Validate
$If\;\left(MAC\_i\left(n^{i}\right)==TRUE\right)$
             Validate
$If\;\left(MAC\_i\left(p\left(n^{i}\right)\right)==MAC\_i\left(pr\left(n^{i}\right)\right)\right)$
             Validate
$If\;\left(B\left(p\left(n^{i}\right)\right)==B\left(pr\left(n^{i}\right)\right)\right)$
      
$pr\left(n^{i}\right)-Preceding\;Node;B\left(p\left(n^{i}\right)\right)-Blocked\;identifiers,$
        $MAC_{i}-Wireless\;Physical\;Address\;of\;sensor\;node$

5. Start rule-based classification function,          
$C^{RF}=\sum_{i=1}^{N}f(c^{1},c^{2},c^{3}\ldots \ldots c^{N})\forall \;C(AX^{i})$
6. Set event classifier logs for each node 7. Update the logs for each session 8. Redo for all channels

End of Procedure (D)


The rule-based attack classifiers analyze the sensor node’s request at the receiver node depending on successful IHF access by any attacker node. Usually, the legitimate sensor nodes are restricted from accessing IHF as they have been known to interleave functions. From the technical discussion of the proposed model, the novel wireless IHF techniques are identified as more reliable security solutions [51,52,53]. Anyhow, the absence of confidentiality, authentication, and channel-sealing policies creates channel vulnerabilities against crucial wireless attacks. In this circumstance, the lightweight encryption algorithms and authentication algorithms hide the multi-channel data transmissions [54,55]. The proposed system develops channel-aware Advanced Encryption Standard (AES) and Elliptic Curve Cryptography with Digital Signature (ECCDS) algorithms to protect the data on the channel.

Cipher Frames,

(10) $$C^{f\left(i\right)}=s.E\left(Data^{f},A\_Key^{128}\right)\forall C(AX^{i})\;$$

Decrypted Frames,

(11) $$Data^{fi}=s.D\left(C^{f\left(i\right)},A\_Key^{128}\right)\forall C(AX^{i})$$

$$A\_Key^{128}-AES\;Key\;$$

$$s-Random\;blocks\;taken\;by\;AES\;\alpha \;e^{AX},\;energy$$

Equations (10) and (11) provide the details of channel frame encryption and decryption models support for secure data transmission. The data confidentiality on wireless channels is achieved by blocking specific AES functions. In the same manner, the participant authentications are ensured with the help of ECCDS principles. As sensor nodes are limited to energy resources and other memory constraints, device-specific authentication principles are required. Based on this issue, ECCDS has been loaded inside the sensor node to authenticate insiders and outsiders. Equation (12) provides the technical aspects of ECCDS with message authentication codes.

(12) $$A\left(n\right)=ECCDS\left(session_{id}\left|\left|m\left(n\right)\right|\left|t\left(s\right)\right|\right|nc\right)$$

$$m\left(n\right)-message\;authentication\;code\;$$

$$t\left(s\right)-session\;timestamp$$

nc−Random nonce

**Figure 7:**
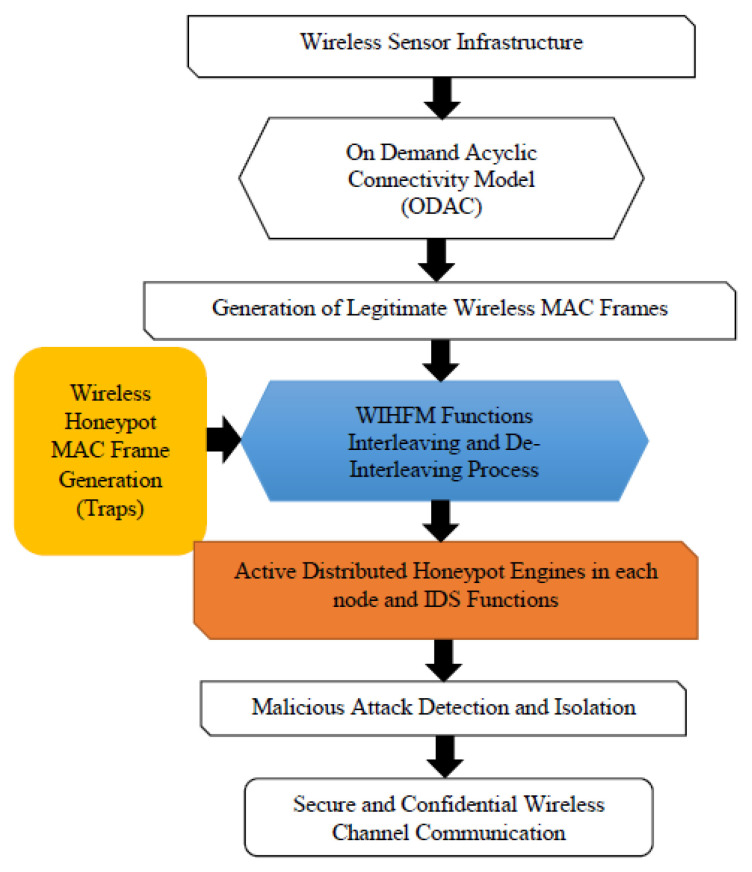
Design of WHIFM Phases.

Thus, the proposed security principles and wireless IHF models protect vulnerable WSN channels from attackers. Figure 7 illustrates the overall proposed design of WIHFM and its internal phases. Section 3 provides an experimental circumstance and the performance benefits of existing and proposed techniques.

## 3. Results and Discussion

The experimental test has been created based on the technical specifications listed in Table 1. As per the configured network environment, data communication is initiated through multi-hop channels. In this neighbor-based mode of data communication, each sensor node shall act as either the source, destination, or forwarding node. The WSN has been developed with 300 sensor nodes at the maximum population case [56,57]. As mentioned in the simulation specifications, the proposed WIHFM and ODAC provide major contributions to virtual wireless channel management policies and IHF policies, respectively. At the same time, the experiment has been implemented with a routing fusion of an Ad hoc On-Demand Distance Vector (AODV)-routing protocol and Optimal Link State Protocol (OLSR) to handle multi-channel routing procedures. In this experiment, the WSN nodes are configured with a random mobility model that attains a velocity rate between 10 m/s (m/s) and 50 (m/s). The experiment allows both legitimate network nodes and attacker nodes to build vulnerable channel conditions [58,59].

As the simulation environment has been created for a worst case WSN scenario, in the configured geographical area (1000 m × 1000 m), the deployment of 300 sensor nodes with a random mobility model create appropriate complexity levels in terms of population, channel establishment time, and link management. Generally, the maximum transmission range of a sensor node is configured up to 100 m. In this scenario, the transmission range has been increased up to 150 m to acquire the actual performance of a real-time sensor environment. In this case, a constant position mobility model can also be used for deploying WSNs. However, the mobility model leads to network uncertainties and random options for constructing the links and channels for each iteration in the simulation model. This practice helps to observe the performance of the proposed system under complicated geographical conditions rather than constantly deployed sensor nodes.

**Figure 8:**
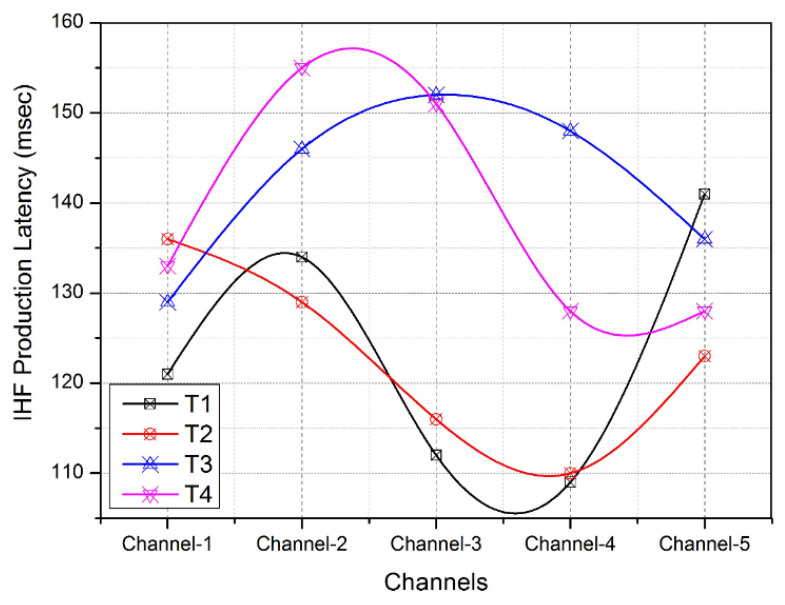
IHF Production Latency.

Here, the proposed experimental setup expects a wormhole attacker, a packet-dropping attacker, an identity theft attacker, a MAC frame-eavesdropping attacker, and a misrouting attacker. Apart from legitimate sensor nodes, attacker nodes are created in order to initiate the attacks on various channels. These attacker functions are called from selected attacker nodes to initiate malicious events randomly, as per the simulation setup. The wormhole nodes (calling functions to record the neighbor node’s data and tunnel them into another node) are created around legitimate sensor nodes in the network. The packet-dropping attacker node calls the functions to collect the data from a legitimate node and drop the same data at the receiving point itself. In this simulation, the identity theft attacker is considered to initiate the functions to obtain the neighbors’ identifiers and manipulate these identifiers due to the attacker’s lack of network reputations. The eavesdropping attacks in the sensor networks are configured with the simulated functions for recording the nearest data transmissions of any node. Finally, the misrouting attack calls the simulated functions that change the legitimate routing table entities into fraudulent entities. The legitimate sensor nodes shall expect any attack from other nodes to be intercepted by the wireless channel. The network simulation arrangement has been deployed using Network Simulator (NS-3.35) tool. In this work, we have implemented network setup and data transmission scenarios using the C++ options of NS-3. Similarly, the internal functions of the existing techniques and proposed models were created with the help of python library files. The proposed model has been developed and compared with the notable existing techniques such as SZ-MAC, BASR, and TBNE. The proposed work and the works acquired for the experimental comparison are implemented in the NS-3 tool under the specified network environment (Table 1) and network features. This evaluation identifies the contributions of each work against uniform network natures and provides a reasonable outcome. Figure 8 exemplifies the production latency (milliseconds (msec)) of WIHF in the wireless channel. The transformation of IHFs into legitimate frames in the wireless channel employs reasonable latency values at different sessions:T1, T2, T3, and T4.

**Figure 9:**
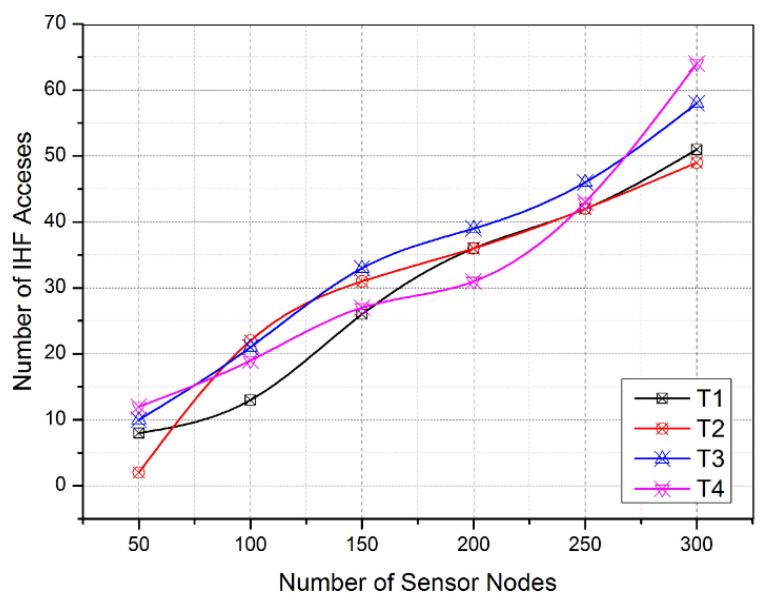
Number of IHF Accesses.

Each session consumes a unique communication duration based on the currently available communicating parties in the network [60]. As given in Figure 8, the IHF production latency varies from 110 milliseconds (msec) to 155 msec. The observation of the IHF production latency denotes the average time taken to produce and insert the IHFs into a legitimate MAC frame sequence on the channel. According to the experiment, Figure 8 shows the average IHF production latency for each channel (five channels are observed). Similarly, Figure 9 depicts the unknown access into the IHF traps on the channel. As per the evaluation, the number of malicious injections over the IHFs gradually increases due to various channel attacks, as mentioned earlier. These malicious nodes are being caught in IHF traps continuously and being misled into distributed wireless honeypot engines running in each sensor node [61,62].

**Figure 10:**
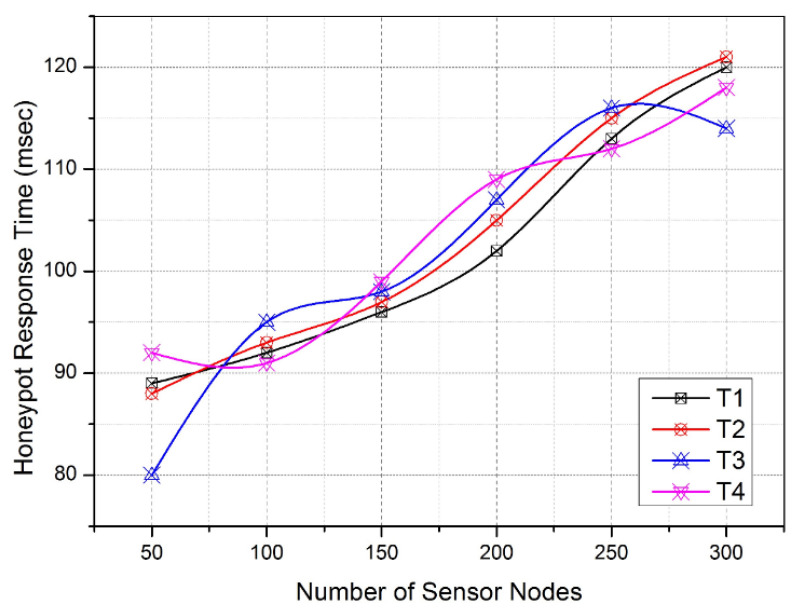
Honeypot Response Time.

Figure 9 illustrates the increasing number of sensor nodes from 50 to 300 at the different iterations of the simulation. On the other side, the changing communication sessions T1, T2, T3, and T4 receive frequent attacker requests to intercept the channel activities that are identified and isolated in order to distribute the honeypot environment. In this manner, Figure 8 and Figure 9 provide the IHF production latency and the benefit of using IHF traps, respectively. Consequently, the activation of the honeypot engines and their response time play a crucial role in ensuring reactive intrusion detection activities in the WSN. As per the necessity, Figure 10 signifies the average honeypot response time taken by the sensor nodes under the proposed WIHFM. Here, the response time varies between 80 msec and 120 msec. The variation in the response time depends on the idleness rate or multiprocessing rate of each sensor node at time ‘T’.

As shown in Figure 10, the response time is slowly increasing as the number of sensor nodes increases at the iterative slots. The increasing number of nodes increases the overall channel liveliness and multiprocessing heaps.

**Figure 11:**
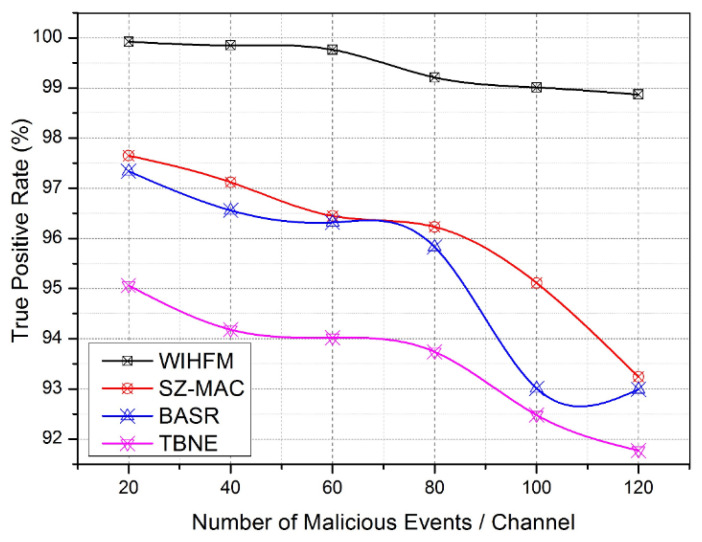
True Positive Rate.

Based on these discussions, the practical abilities of the proposed WIHFM are recognized during different iterations. The functional aspects of the proposed WIHFM are compared with SZ-MAC, BASR, and TBNE. Each existing technique uses unique channel security principles such as secure MAC protocol, blockchain commutations, and key-based trust evaluation models, respectively. In this regard, Figure 11 depicts the average true positive rate of different techniques throughout the alteration of several malicious injections into the channel.

**Figure 12:**
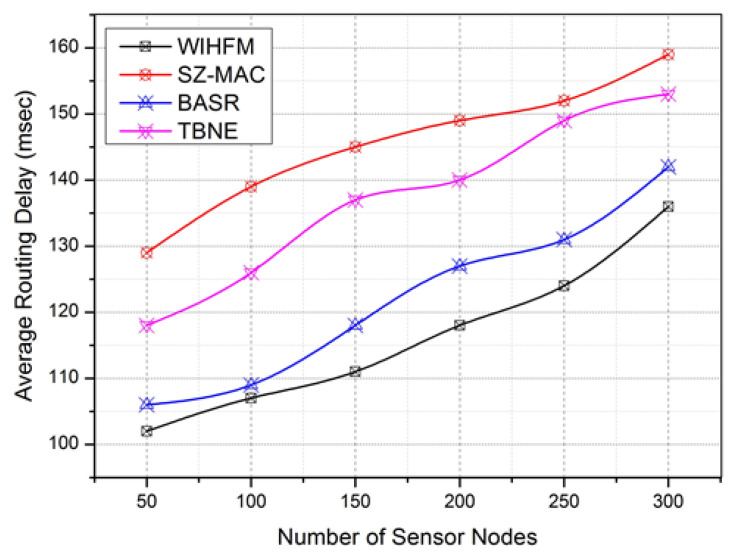
Average Routing Delay.

Compared to other channel security models, the proposed WIHFM creates complex random honeypot trap possibilities uniquely [63]. Consequently, malicious events are widely trapped under IHFs. The malicious events are categorized under false data injections, wormhole starts, packet drops, identity hacks, and other channel intrusions through MAC frames. Figure 11 shows the proposed model’s true positive rate between 89.8% and 99.6%. At the same time, the existing techniques achieve a rate of true positives between 91.6% and 97.6%. Figure 12 clarifies the average routing delay for each data transmission during vulnerable channel activities. As discussed, the proposed model has a better honeypot management system with limited latency rates. Additionally, the fusion of AODV and OLSR enables fast route updates during attacker interactions.

In this routing strategy, the AODV optimizes the reactive route updates and OLSR optimizes overall network updates latencies. Based on distributed WIHFM functions, the need for optimal routing fusion confirms a minimal routing delay compared to other existing techniques. In this scope, a secure and trusted routing protocol is another choice for notable research works [64,65].

**Figure 13:**
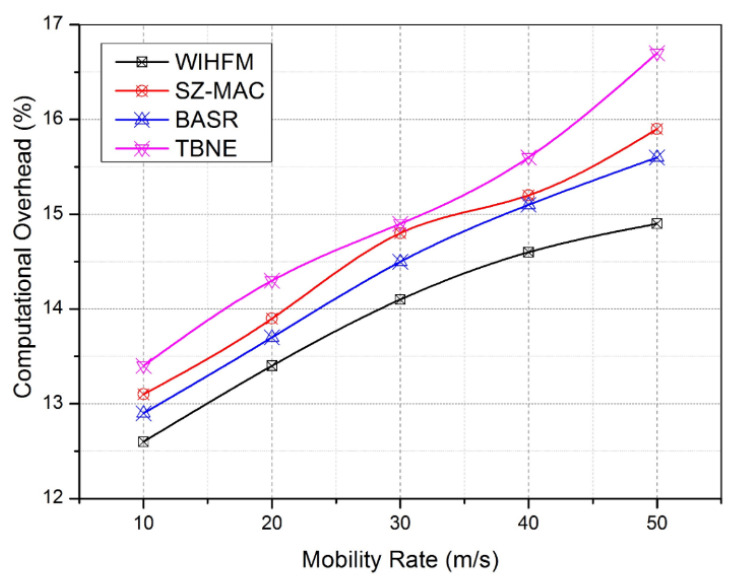
Computational overhead.

In this experiment, the proposed WIHFM and BASR produce almost bordering routing delay (msec). However, the proposed WIHFM has a smaller routing delay than BASR between 102 msec and 127 msec. Other techniques attain a maximum routing delay between 153 msec and 160 msec. This result shows the optimal handling of data routing through multiple channels. Figure 13 delivers the additional computation burden incurred by each node during the overall WIHFM functions and other existing security functions.

**Figure 14:**
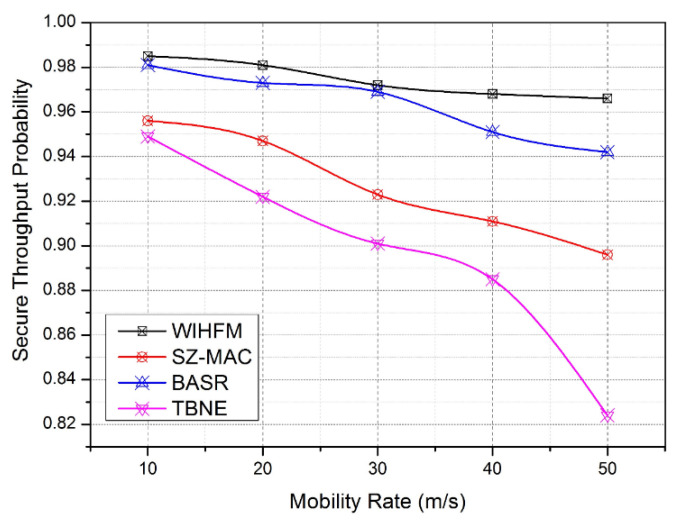
Secure Throughput Probability.

The proposed functional internals of each security technique consume significant computation overhead from 12.6% to 16.8%. In these cases, the proposed model achieves a slightly improved overhead compared to other techniques between 12.6% and 14.9%. Similarly, BASR attains its maximum overhead of 15.6%. Compared to other security techniques, the proposed model has lightweight IHF procedures, distributed honeypot management principles, and attack detection rules in each node. Hence, the proposed WIHFM manages the finest computation overhead from among the other systems, as the velocity of the sensor node varies from 10 m/s to 50 m/s. In the same manner, the important feature of the proposed security framework is examined using a secure throughout probability rate as given in Figure 14. Secure throughput is defined as the rate between the protected frame data and overall data transmitted through the wireless channel. Here, TBNE and SZ-MAC attain a lower secrecy rate as these techniques are built with insufficient resilient node evaluation procedures. Subsequently, these techniques secure the channel frames at the minimal rates of 0.82 and 0.88, respectively. As the node’s velocity changes from 10 m/s to 50 m/s gradually, the secrecy management hurdles increase for all techniques on the vulnerable channels. Moreover, the proposed model maintains the lowest secure throughout probability rate as 0.96 against the maximal movement of the sensor node. This experiment justifies the ability of the overall network security level provided by the proposed WIHFM during uncertain node movements.

Complexity evaluation experiments and encryption quality analysis tasks are essential for optimizing the performance of tiny wireless sensor nodes. Table 2 shows the complexity levels of various encryption mechanisms and the device adaptability solutions.

As the proposed method uses AES and ECCDS for establishing channel confidentiality and neighbor authentication, respectively, Table 2 provides an understanding of cryptography complexities. According to the experiments, AES/ECCDS fusion takes 127 KB in the node’s memory and 515 msec of latency. In contrast, other approaches such as the Data Encryption Standard/Rivest Shamir Adleman (DES/RSA), AES/ Digital Signature Algorithm (AES/DSA), Twofish/DSA, and Diffie–Hellman (DH)/ECCDS have considerable complexities under time and space domains. According to the complexity analysis, the AES/ECCDS suite is identified as a compatible technique for tiny sensor nodes.

Likewise, Table 3 provides the working strategies and response times for the various cryptography suites. As per the identifications, the response time of AES/ECCDS is a minimum of 485 msec compared to other suites. In this regard, the lightweight encapsulation adaptability of the AES/ECCDS suites is better for the sensor node platform with respect to improving the channel security. This experiment reveals that other suites achieve response times between 638 msec and 780 msec as maximum quantities compared to the AES/ECCDS suite. The importance of virtual link management functions has been evaluated, as shown in Table 4. The observations of Table 2, Table 3 and Table 4 are extracted from the simulator to evaluate the complexity levels of the channel security mechanisms. The details do not replicate any other sources. The purpose of this complexity evaluation is to justify the choice of the appropriate security frameworks for resource-limited wireless sensor networks.

**Figure A1:**
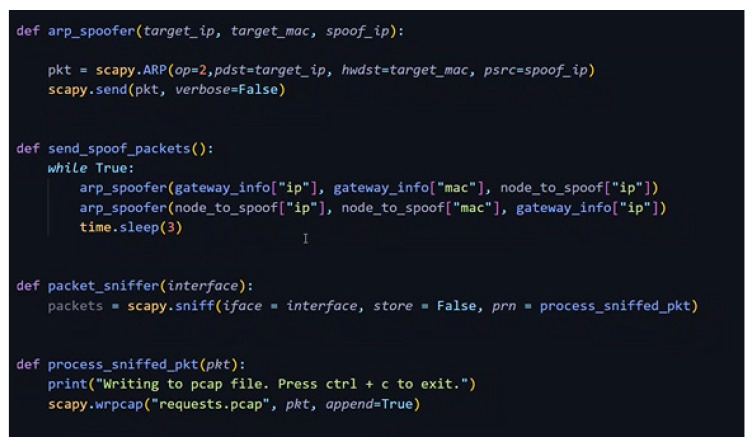
Python snippet for attack packet spoofing function.

In this evaluation, the proposed WIHFM-ODAC system achieves an optimal channel recreation time (msec) during attacker interruptions (Figure A1) and other channel interruptions. At the same time, BASR, SZ-MAC, and TBNE consume more channel recreation time, which disturbs the overall network liveliness. Consequently, the rate of channel availability reduces frequently for all sessions in the network. As the ODAC has on-demand connectivity establishment functions based on dynamic graph computations, the sustainability of each wireless channel is recreated more optimally than in other models. In this experiment, the specificity metric plays an important role in determining the capability of the system in order to show the correctly measured legitimate frames. In particular, the system specificity is defined as $tn/\left(tn+fp\right)$. In this metric, tn−true negative; fp−false positive.

Table 5 illustrates the observed specificity rates (%) of the proposed models and existing techniques. The better performance of the proposed model against the existing techniques is attained through a secretly interleaved frame analysis and distributed honeypot model activation procedures.

**Figure A2:**
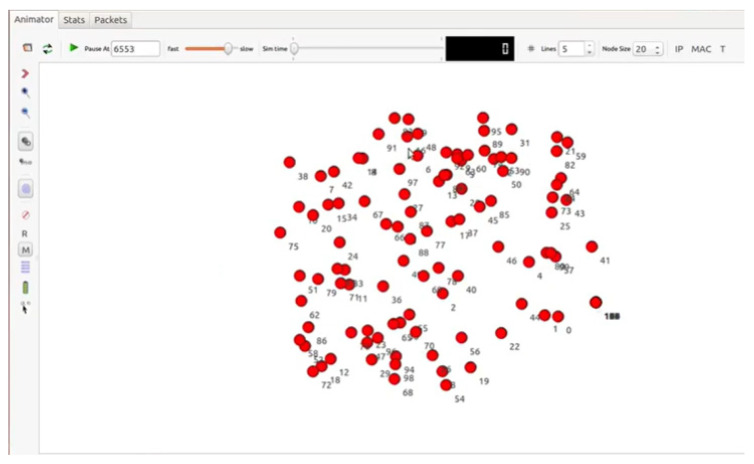
NS-3.35: Network Scenario (100 Sensor Nodes).

**Figure A3:**
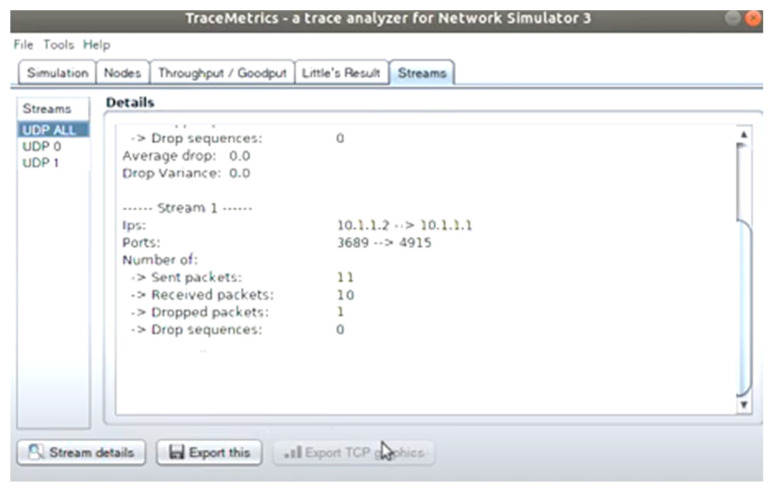
NS-3.35: Data Transmission.

In this manner, the resiliency of ODAC has been justified. Thus, the proposed WIHFM ensures secure wireless communication through significantly developed IHF functions (Figure A2 and Figure A3). In addition, the proposed WIHFM can be applied for the benefits of moderate security platforms and applications. Specifically, the application of WSNs in home automation systems and agriculture systems requires moderate or minimal security considerations [66,67]. Under these environments, the proposed WIHFM and distributed security policies defend against outsiders’ or attackers’ intrusion into any network device (sensor node). Notably, the performance of WIHFM for minimal security applications inevitably reaches the maximum specificity rate and sensitivity rate. At the same time, the sensor nodes adopted for agricultural networks and home automation networks require suitable computation capabilities to achieve the maximum secure throughput rate through the channels. In addition, the rate of computation efficiency and power efficiency decides the successful execution of WIHFM’s procedures in each sensor node. Consequently, the application of the proposed WIHFM needs environmental validation processes and configuration validation processes to ensure optimal energy consumption, latency, throughput, sensitivity, and specificity in real-time.

## 4. Conclusions

The security principles and cryptography mechanisms used for protecting data communications are widely practiced among various types of wireless networks. However, the identification of a novel MAC security management system supports resilient WSN protection against various attackers. In this article, the proposed system was implemented with distributed WIHFM and ODAC virtual link management features. As per the newly developed model, the IHF features were identified as novel distributed solutions implemented in each sensor node of the WSN. Similarly, the WIHFM enabled IHF computations and distributed wireless honeypot engines to create random attacker traps to coax the attackers into forged honeypot resources of the sensor node. Based on the proposed model, the attackers and the malicious activities were effectively trapped in IHFs. Consequently, the trapped accesses were recognized as either intrusions or attacks. Finally, the proposed model was compared with the existing frameworks such as BASR, SZ-MAC, and TBNE, as shown in the experimental section. Moreover, the proposed WIHFM outclassed other techniques, as discussed through various performance metrics. However, the proposed WIHFM has limitations regarding its uncertain IHF latency and honeypot response time as the network dynamics change continuously. Future technical aspects, such as the intelligent network dynamic analysis model, dynamic programming model, and uncertainty computation model, are expected to compensate the limitations of WIHFM.

As the current network MAC models are not optimally enriched with low-powered dynamic programming solutions against channel uncertainties, the need to improve the static limitations of WIHFM is required in the future. In addition, the next-generation honeypot solutions in the wireless environment are expected to be improved with these distributed trap management policies for application-specific sensor platforms. Notably, the next-generation WSN models are widely sensitive to the energy utilization rate with respect to their application platform. Considering the internals of notable WSNs’ applications, the proposed novel WIHFM can be implemented for military sensor networks, healthcare sensor networks, and other deployment strategies exclusively in the future.

## Figures and Tables

**Table 1:** Simulation Specifications.

Simulation Parameters	Details
Simulator Name	NS-3.35
Number of Sensor Nodes	Maximum 300
Network Area	1000 m × 1000 m
MAC	IEEE 802.11
Channel Type	Wireless
Virtual Backbone	ODAC
MAC Security	WIHFM
Data Traffic	Variable Bit Rate (VBR)
Signal Propagation	Two Ray Ground
Initial Energy (Joules)	50
Transmission Range (meters)	150
Channel Frequency (GHz)	2.4
Mobility Rate (m/s)	10, 20, 30, 40, 50
Antenna Model	Omnidirectional
Routing Protocol	AODV/OLSR
Simulation Time (Seconds)	300

**Table 2:** Complexity Levels and Device Adaptability.

Encryption Techniques	Space Complexity (Kilobytes, KB)	Time Complexity (Milliseconds)	Device Adaptability
DES/RSA	178	890	Not suitable for tiny sensors
AES/DSA	166	655	Moderately good for tiny sensors
Twofish/DSA	172	660	Moderately good for tiny sensors
DH/ECCDS	170	726	Not suitable for tiny sensors
AES/ECCDS	127	515	Suitable for tiny sensors and patch-type devices

**Table 3:** Response Time and Security Strategy.

Security Techniques	Working Strategy	Response Time (Milliseconds)	Lightweight Encapsulation Adaptability
DES/RSA	Symmetric/Authentication	780	No
AES/DSA	Symmetric/Authentication	638	No
Twofish/DSA	Symmetric/Authentication	645	No
DH/ECCDS	Asymmetric/Authentication	704	No
AES/ECCDS	Symmetric/Authentication	485	Yes

**Table 4:** Dynamic Channel Recreation Strategy.

Backbone Management Strategies	Channel Recreation Latency (msec)
WIHFM-ODAC	87
BASR	126
SZ-MAC	137
TBNE	155

**Table 5:** System Specificity.

Backbone Management Strategies	Specificity (%)
WIHFM	98.95
BASR	95.08
SZ-MAC	95.34
TBNE	94.56

## Data Availability

Not Applicable.
